# Quality of small incision cataract surgery

**Published:** 2015

**Authors:** William H Dean

**Affiliations:** Ophthalmologist: Bristol Eye Hospital, Bristol, UK.

**Figure:**
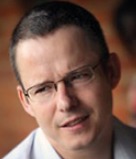
William H Dean

Cataract is the most common cause of blindness. Cataract extraction with intraocular lens implantation is one of the most frequently performed and most effective surgical procedures in the field of medicine, worldwide.

Small-incision cataract surgery (SICS) is also called manual small-incision cataract surgery (MSICS) or sutureless extra-capsular cataract extraction (SECCE). It is a safe, cost-effective procedure with very good outcomes. The technique is well described in the literature, as is the management of its complications.

The quality of SICS, and most importantly the outcome for the patient, can be excellent. The World Health Organization (WHO) advises aiming for post-operative outcomes of at least 80% good presenting vision or at least 90% with best-corrected vision, and this is attainable with SICS. But with such a good procedure at your fingertips, how do you aim for and achieve quality? And how do you plan for, maintain, and monitor the best possible outcomes for your patients?

## Training and learning

It is important to learn, to want to learn, and to maintain a good technique. There are many good resources available.

- MSICS classroom: http://classroom.globalsight.org
- Eye Surgery in Hot Climates 4th Edition (JP Publishers, 2015).
- Standard Operating Procedure Manual for Modern Small Incision Cataract Surgery (Tilganga Eye Centre): available via the Global Sight Alliance website: www.globalsight.org
- Aravind Eye Hospitals’ book Manual Small Incision Cataract Surgery: available as a free iBook download from iTunes: http://tinyurl.com/pu757w7
- Sutureless ECCE (2nd Edition) video: www.youtube.com/watch?v=LszyZqqR5v4

Time spent reading and watching videos again and again is time well spent. However, a good teacher is critical. It is important to be able to practise and get feedback. There is a big role for simulation in training and practice – either ‘wet-lab’ as in the use of animal eyes to learn and practise; or ‘dry-lab’ as in the use of artificial eyes and simulators (HelpMeSee.org and simulatedocularsurgery.com).

## Evaluation and selection for surgery

As a surgeon, you may not see all of your cataract patients before surgery. If this is the case, it is imperative that the nurses working with you are diligent in their pre-operative assessment. All patients should have the following assessments: visual acuity, intra-ocular pressure (IOP), pupils (looking for a relative afferent pupillary defect, or RAPD), and past ophthalmic history. Selection for surgery is very important. Listing a patient for routine cataract surgery who has an RAPD, high IOP, ora history of severe retinal disease may lead to a poor outcome. The patient should be fully assessed, and appropriate consent taken before a ‘guarded prognosis’ is made. In addition, for good vision outcomes, it is essential to have accurate biometry and to have a large range of intraocular lenses available.

## Surgical technique

The procedure itself is wonderful when all goes well; however, every eye is different, every operation is different, and each and every step of the procedure is as important as the one before.

A good draping is necessary to capture the eyelashes, especially those of the upper lid.

The superior rectus suture is important: it immobilises the eye and assists with scleral tunnel ‘opening’ when extracting the nucleus. Going too deep with the needle may penetrate the eye, and going too shallow will engage the conjunctiva only.

**Figure 1.**
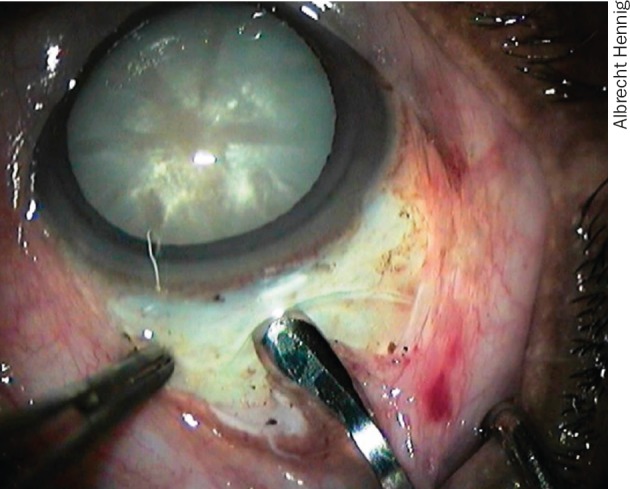
Stabilising the sclera with toothed forceps while making a frown-shaped incision

Firm scleral fixation (throughout the tunnel construction) should be maintained by using good forceps (Figure 1).

The scleral tunnel is very important. A very curved and ‘frown-shaped’ incision should be made initially. If the incision is too flat, this will induce a significant against-the-rule astigmatism. Use adequately sharp blades.

When forming the tunnel with the crescent blade, aim to see just enough of the metal of the blade. If you cannot see any of it, you are too deep and will likely prematurely enter the anterior chamber. If you see too much, a buttonhole will form. Dealing with complications in tunnel construction may be necessary: a button hole may lead to leakage and should be undermined in a different plane or a new entry site fashioned. Premature entry into the anterior chamber will often require a suture.

The capsulotomy can be linear or continuous-curvilinear. Lots of small puncture marks are necessary for a linear capsulotomy. Aim for just above the halfway line. This will leave a good inferior portion to protect the corneal endothelium when extracting the nucleus, but also enough of a superior portion to support a sulcus-placed IOL if the posterior capsule is ruptured and cannot support an IOL.

Thorough hydro-dissection helps mobilise the nucleus. Always check that the cannula is on tightly before entering the eye. Lift the capsule slightly when injecting underneath it.

The most difficult part of the procedure is the mobilisation of the nucleus. Once you are happy that the nucleus is free in the bag, inject visco-elastic into the anterior chamber to protect the endothelium. Use the cannula, while slowly injecting visco-elastic, to dislodge the upper equator of the lens nucleus. The important point is to press backwards and slightly down within the scleral wound **beyond** the upper equator, such that the upper part of the nucleus actually starts to move **forward** rather than backwards.

Inject a good amount of visco-elastic behind the nucleus to push back the posterior capsule before inserting the vectis or fishhook needle to extract the nucleus.

Once the nucleus is removed, take great care when removing the soft cortical lens matter. Increase the magnification on the microscope for this stage, as well as for the capsulorhexis.

An injection of antibiotic into the anterior chamber (intra-cameral) should be performed at the end of the procedure (with either cefuroxime (1 mg in 0.1 ml) or moxifloxacin, but **only** if you can guarantee that the concentration will be correct every time. This may help to prevent postoperative endophthalmitis, but can severely damage the corneal endothelium if an incorrect dosage is injected.

## Complications and their management

Dealing with vitreous loss is important. It is most important that no vitreous remains in the anterior chamber going up to a wound or paracentesis. This is certain to invite future infection, chronic macular oedema, or even a retinal detachment. It should almost always be possible to place either a posterior chamber or sulcus IOL. Avoid an anterior chamber IOL if at all possible.

If there is any doubt about the wound, or if the initial incision is too flat, then place a 10-0 nylon suture in the main incision.

## Post-operative treatment and evaluation

Patients must be given a combination of antibiotic and steroid drops for a few weeks after surgery. It is important to ensure that patients understand the importance of using the drops, and also how to use them.

Post-operative evaluation is critical. Examine patients on the first postoperative day to diagnose and treat surgical complications. Vision assessment and ideally refraction should take place at 4–6 weeks after surgery and are essential for assessing surgical outcomes. Patients and/or carers must be educated about possible complications. They must be told what to do and where to go if they have untoward symptoms. This will help to ensure early diagnosis and treatment of complications such as intraocular infection.

## Audit

The value of being interested in, recording, monitoring and then reflecting on all your surgical outcomes cannot be over-emphasised. On a very personal level, as a surgeon, this will improve your selection, technique and management; ultimately, it will make you a better surgeon and improve outcomes for the patients that you treat. Audit of outcomes should be absolutely mandatory everywhere. It may seem like an intrusion or extra effort, but it definitely makes you a better surgeon.

## Conclusions

Ultimately, the best surgical safety, technique, and outcomes for your patient are not up to a textbook, DVD, website or trainer. They are in your, the surgeon's, hands. Only you can strive for the best surgical outcome, and the eye team can help you to achieve this.
